# Causal associations between the gut microbiota and multiple myeloma: a two-sample Mendelian randomization study

**DOI:** 10.3389/fnut.2024.1400116

**Published:** 2024-06-14

**Authors:** Chun-Yan Zhang, Dong Zhang, Wen-Rui Sun, Hai-Long Tang, Biao Tian, Li-Hong Hu, Wu-Yue Hu, Ya-Ya Gao, Miao-Yu Li, Wan-Ting Xiao, Shan Gao, Guang-Xun Gao

**Affiliations:** ^1^Department of Hematology, Xijing Hospital, Air Force Military Medical University, Xi’an, China; ^2^Department of Plastic Surgery, Tangdu Hospital, Air Force Military Medical University, Xi’an, China

**Keywords:** gut microbiota, multiple myeloma, Mendelian randomization, nutrition, dietary

## Abstract

**Background:**

Previous observational studies have indicated a potential association between the gut microbiota and multiple myeloma (MM). However, the relationship between the gut microbiota and MM remains unclear. This study aimed to ascertain the existence of a causal link between the gut microbiota and MM.

**Methods:**

To investigate the potential causal relationship between gut microbiota and MM, a two-sample Mendelian randomization (MR) analysis was conducted. Exposure data was obtained from the MiBioGen consortium, which provided genetic variants associated with 211 bacterial traits. MM outcome data was obtained from the FinnGen consortium. The selection of Single nucleotide polymorphisms estimates was performed through meta-analysis using inverse-variance weighting, and sensitivity analyses were conducted using weighted median, MR Egger, Simple mode, and MR-PRESSO.

**Results:**

The results of the study demonstrated a significant positive correlation between the genus *Eubacterium ruminantium* group and the risk of MM (OR 1.71, 95% CI 1.21 to 2.39). Conversely, the genus: Dorea (OR 0.46, 95% CI 0.24 to 0.86), Coprococcus1 (OR 0.47, 95% CI 0.22 to 1.00), RuminococcaceaeUCG014 (OR 0.57, 95% CI 0.33 to 0.99), *Eubacterium rectale* group (OR 0.37, 95% CI 0.18 to 0.77), and order: Victivallales (OR 0.62, 95% CI 0.41–0.94), class: Lentisphaeria (OR 0.62, 95% CI 0.41 to 0.94), exhibited a negative association with MM. The inverse variance weighting analysis provided additional support for these findings.

**Conclusion:**

This study represents an inaugural exploration of MR to investigate the connections between gut microbiota and MM, thereby suggesting potential significance for the prevention and treatment of MM.

## 1 Introduction

Multiple myeloma (MM), which is the second most prevalent hematological malignancy, exhibits considerable heterogeneity. It is characterized by the presence of malignant plasma cells that excessively produce monoclonal immunoglobulin paraproteins within the bone marrow (1). Typical symptoms in MM patients include hypercalcemia, renal impairment, anemia, and bone abnormalities. MM is associated with a range of risk factors encompassing lifestyle, genetics, diet, occupation, environment, and notably inflammation (2). However, the precise etiology and pathogenesis of this disease remain largely elusive. Despite the discovery of novel medications that augment treatment options for MM patients, achieving a complete cure remains a formidable challenge due to drug resistance and relapse (3). Consequently, it is crucial to investigate the origins of MM to impede its progression and promote effective therapeutic strategies.

The gut microbiota, which consists of microbes including bacteria, fungi, viruses, and protozoa, is considered as the largest symbiotic microbial community within the human body (4). It plays a vital role in numerous physiological processes, such as the absorption of nutrient metabolites, immune system regulation, supporting of blood cell formation, and influencing neurobehavioral characteristics (5, 6). Recent research indicates that the gut microbiota significantly impacts the development, treatment response, and overall progression of various cancers, particularly through its interactions with the tumor microenvironment (7). The microbiota could influence the bone marrow microenvironment by generating bioactive metabolites such as short-chain fatty acids (SCFAs). These SCFAs have the potential to inhibit nuclear factor kappa-light chain enhancer of activated B cells (NF-κB), interleukin-6 (IL-6), and tumor necrosis factor alpha (TNF-α), while potentially promoting an increase in IL-10, T helper 17 cells, and Th1 cells (8). Recent evidence suggests that the microbiota plays a role in the progression of MM by promoting inflammation. Notably, certain microorganisms, such as Streptococcus and Klebsiella, which possess nitrogen-reusing capabilities, were found to be significantly elevated in MM patients and contributed to disease progression when compared to healthy individuals (9). Additionally, a previous study proposed that the presence of Th17 cells in the intestinal tract, facilitated by *Prevotella heparinolytica*, may enhance their migration to the bone marrow, thereby potentially contributing to the advancement of MM (10).

Although a cure for MM remains elusive for most individuals, ongoing advancements in treatments have shown promise in extending lifespan, and a specific subset of patients may even achieve a cure. Recent studies indicate that dietary habits and lifestyle factors can significantly impact the health and longevity of individuals with MM (11). The gut microbiota plays a crucial role in the absorption and metabolism of dietary nutrients. By modifying the microbiota, metabolome, and immune microenvironment through a beneficial dietary pattern, outcomes in plasma cell disorders, such as progression and survival rates, can be improved while reducing toxicities and comorbidities. Consumption of a nutritionally balanced diet has the potential to improve the composition and diversity of the gut microbiota by increasing the abundance of SCFA generators and reducing the presence of nitrogen-producing and bile-tolerant bacteria. This modulation of the gut microbiota may have implications for slowing the progression of MM (12). However, the relationship between the gut microbiota and MM is still not fully understood.

To investigate the association between exposures and outcomes, Mendelian randomization (MR) is increasingly being utilized as a method to integrate summary data from genome-wide association studies (GWAS). The primary advantage of MR in establishing causality is its capacity to generate instrumental variables (IVs) for exposure by leveraging single nucleotide polymorphisms (SNPs) (13). Consequently, the influence of common confounding factors on the association between genetic variations and outcome is minimized (14). Based on these principles, our initial approach involved a comparative analysis using data from the MiBioGen and FinnGen databases to elucidate the causal link between gut microbiota and MM.

## 2 Materials and methods

### 2.1 Study overview

**Figure 1:**
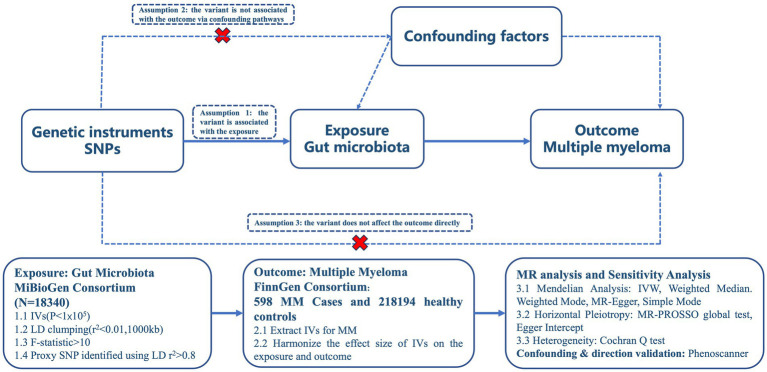
Study overview.

In order to ensure the accuracy of the findings, this study is based on three key assumptions derived from previous research: ① the independent variables (IVs) exhibit a significant correlation with the exposure; ② the IVs are unaffected by any confounding factors; and ③ only exposure to the IVs has an impact on the outcome. These assumptions were cited in the study. The study design adhered to the guidelines provided by two example MR studies (14), as depicted in Figure 1 outlining the analytical framework.

### 2.2 Gut microbiota and multiple myeloma data source

The genetic variations of gut microbial species, specifically SNPs data, were sourced from the largest meta-genome-wide association study on human gut microbiota, known as MiBioGen.^1^ The dataset comprised 18,340 individuals and encompassed 211 taxa from 24 cohorts spanning Europe, North America, and East Asia. To conduct the mapping analysis of microbial quantitative trait loci (mbQTL), adjustments were made for age, gender, study-specific covariates, and genetic principal components (15). For our primary analysis, we utilized the GWAS data for MM obtained from the FinnGen consortium which encompassed 598 MM cases and 218,194 controls without the disease. This publicly accessible data can be accessed on the website.^2^

### 2.3 Genetic instrument variables selection

The accuracy of causal inferences heavily relies on selecting appropriate IVs. Subsequently, SNPs for each taxon underwent filtration through a series of steps: SNPs with GWAS (*p* < 1 × 10^−5^) were retained for sensitivity analysis. To prevent linkage disequilibrium among selected IVs, two uncorrelated SNPs (with LD *r*^2^ < 0.01 and clumping distance of 1,000 kb) were chosen, ensuring that all SNPs are strongly and independently predicted exposures from the published GWAS at genome-wide significance (16). For consistency, gut microbiota and MM GWAS findings were aligned by matching their respective versions and genetic variations. SNPs absent in the outcome dataset were substituted with Proxy-SNPs that exhibited LD *r*^2^ > 0.8. Additionally, incompatible or palindromic SNPs with intermediate allele frequencies were eliminated. To mitigate the impact of unreliable instruments, we evaluated the *F* and *R*^2^ measurements of each SNP to investigate its influence on gut microbiota using the subsequent equation: *F* = [*R*^2^ × (*N* − 2)]/(1 − *R*^2^), *R*^2^ = [2 × *β*^2^ × EAF × (1 − EAF)]/[2 × *β*^2^ × EAF × (1 − EAF) + 2 × SE^2^ × *N* × EAF × (1 − EAF)]. In this context, *N* and EAF represent the sample size and the frequency of the effect allele, respectively. SNPs were assessed using *F*-statistics, and those with *F* values exceeding 10 were disregarded (17).

### 2.4 Analysis of Mendelian randomization and sensitivity analysis

The “TwoSampleMR” (version 0.5.7) packages in R (version 23.6.1) were utilized for conducting Mendelian randomization analyses. Each bacterial taxa present in the gut microbiota was treated as a distinct exposure event. The primary analysis in this study employed IVW to assess potential causal effects of each phenotype on MM risk. Under the assumption of no horizontal pleiotropy, the IVW method provides unbiased outcomes. Furthermore, various sensitivity analyses were conducted to address potential horizontal pleiotropy, including MR-Egger, weighted median, and Mendelian Randomization Pleiotropy Residual Sum and Outlier (MR-PRESSO). The MR-Egger intercept test was applied to assess horizontal pleiotropy, assuming independence between horizontal pleiotropic effects and genetic variant-exposure relationships. A significance level below 0.05 indicates horizontal pleiotropy. The weighted median approach, which selects the median MR estimate as the causal estimate, was used to evaluate the presence of pleiotropy across multiple genetic variants (18). The MR-PRESSO global test was employed to detect outliers and correct for overall horizontal pleiotropy, ensuring robust MR estimates. In cases of heterogeneity among the instrumental variables, outliers were identified and removed for further MR analysis. If inconsistencies were observed in the estimated causal effects for a specific trait, instrumental variables were reassessed using a more stringent genome-wide significance threshold (19). To evaluate potential variability, Cochran’s *Q* statistics were utilized in the IVW method, Additionally, a leave-one-out analysis was conducted to identify and remove any potential outliers that may have an independent influence on the observed causal association. To address potential confounding variables, an additional search was performed on PhenoScanner^3^ to investigate the association between the selected SNPs, which exhibited notable MR estimates in this study, and other risk factors for MM, with a particular emphasis on Reticulocyte count and hemoglobin levels (20). After removing SNPs associated with confounders, causal effects were re-evaluated to ascertain their significance. Statistical power calculations for causal effect estimates were performed using a web-based MR power calculation tool, with a power threshold of 0.8 considered appropriate to reject false null hypotheses (21). Reverse MR employs the same data source as forward Mendelian randomization. In this scenario, the exposure is considered to be MM, while the outcome is identified as SNPs strongly associated with gut microbiota (*p* < 10^–4^).

### 2.5 Statistical analysis

The statistical significance of the MR effect estimate was evaluated using the false discovery rate (FDR) and adjusted using the Benjamini–Hochberg procedure. FDR correction was conducted using the *q*-value procedure, with a threshold of *q*-value <0.1 to control for false discoveries. A connection between the gut microbiota and MM genera was considered indicative if the *p*-value was below 0.05 and the *q*-value was greater than or equal to 0.1 (22). The MR investigation utilized GWAS summary data, which were obtained with ethical approval for each respective GWAS. Published research and publicly available summary data were utilized in this study, and they can be freely downloaded and used without any restrictions as they have been anonymized.

## 3 Results

### 3.1 An overview of instrument variables in gut microbiota

Following the implementation of the genome-wide significance criterion (*p* < 1 × 10^−5^), the processes including harmonization, LD testing, and *F* statistic validation were conducted. As a result, a range of 3 to 22 SNPs were identified as proxies for 211 distinct bacterial taxa. Remarkably, all retained SNPs exhibited *F* values exceeding 10, indicating a significant association between the IVs and their corresponding bacterial taxonomic entities. The pertinent statistical information pertaining to the compiled list of retained SNPs can be found in Supplementary Table S1.

### 3.2 Associations of gut microbiota on MM

**Figure 2:**
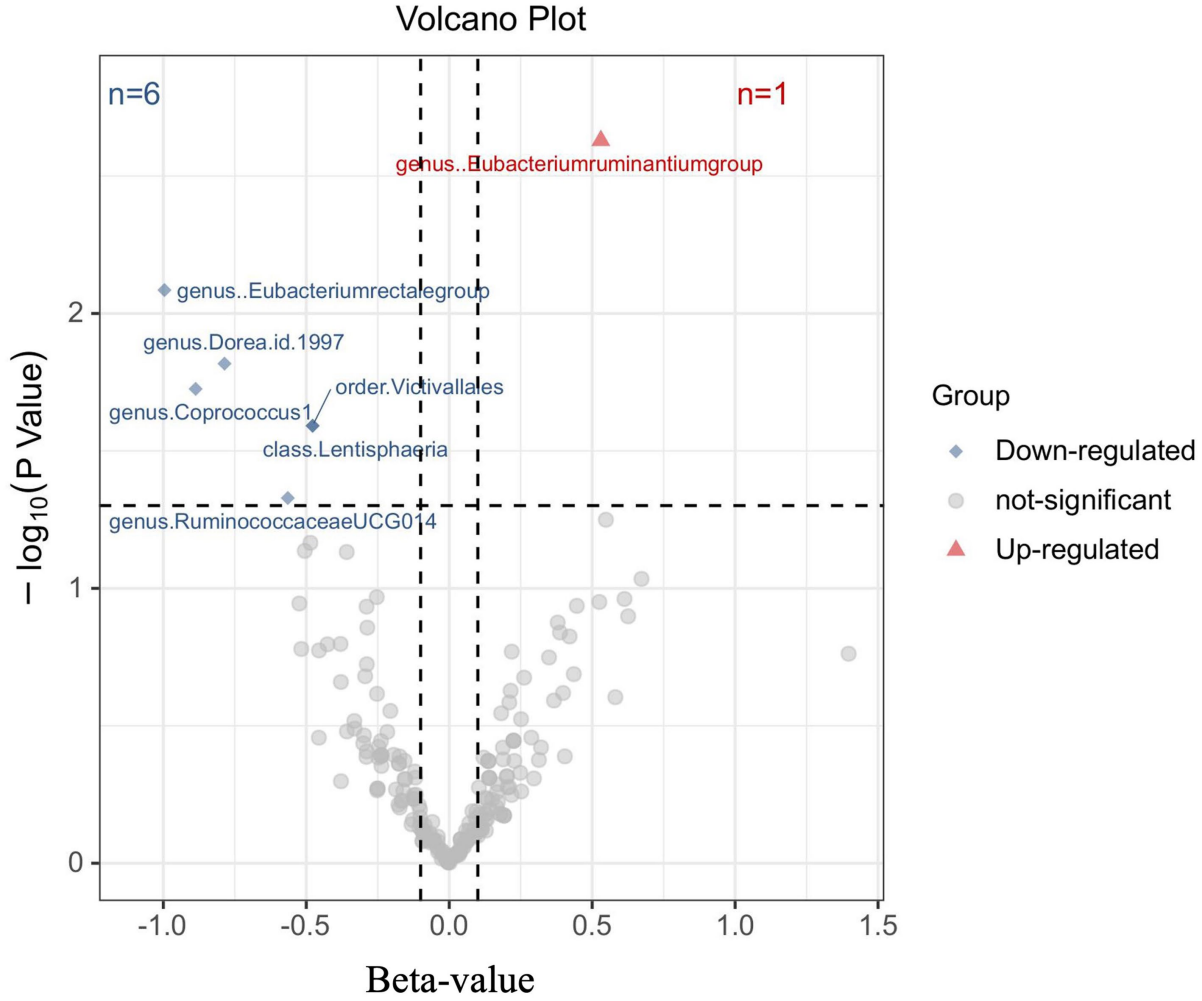
Results of the primary IVW analysis. The volcano plot effectively demonstrates the association between 211 gut microbiota and the risk of multiple myeloma. The *X*-axis represents the beta-value, while the *Y*-axis represents the logarithmic *p*-value with a base of 10. Statistical significance is defined as *p* < 0.05. Notably, the red and blue points on the plot correspond to microbiota genera that pose a risk or provide protection against multiple myeloma, respectively.

To investigate the impact of Gut microbiota on MM, a series of two-sample MR tests were conducted. The initial findings of the MR analysis, pertaining to the causal relationship between 211 gut bacterial taxa and MM, are presented in Supplementary Table S2 and Supplementary Figure S1. By employing the IVW technique and conducting a sensitivity analysis, seven gut microbiota were identified to have strong causal associations with MM (Figure 2). Additionally, it was observed that the presence of the *Eubacterium ruminantium* group is positively correlated with an increased likelihood of MM (IVW OR = 1.7, 95% CI 1.21–2.39, *p* = 0.002).

The causal connection was further confirmed by the Weighted median analysis, which showed a significant association (OR = 1.81, 95% CI 1.12–2.92, *p* = 0.016). The Weighted mode, MR-Egger, and Simple mode analyses also consistently supported this finding; however, statistical significance was not always reached or only reached nominal significance (Supplementary Table S3). This suggests that the presence of the *Eubacterium ruminantium* group could elevate the likelihood of MM. Sensitivity analysis did not reveal any indications of heterogeneity or horizontal pleiotropy.

**Figure 3:**
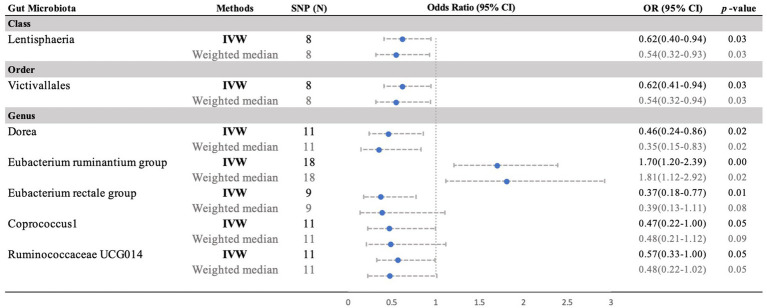
Forest plot of the MR analyses for the associations between gut microbiota genera and risk of periodontitis. CI, confidence interval; MR, Mendelian randomization; OR, odds ratio; SNP, single nucleotide polymorphism; IVW, inverse-variance weighted.

Moreover, negative correlations between six additional gut microbiota and MM were established based on the IVW analysis, showing nominal significance (*p* < 0.05, Supplementary Table S3) for these six phenotypes. As depicted in Figure 3, the genus: Dorea (IVW OR = 0.46, 95% CI 0.24–0.86, *p* = 0.015), genus: Coprococcus1 (IVW OR = 0.47, 95% CI 0.22–1.00, *p* = 0.049), genus: RuminococcaceaeUCG014 (IVW OR = 0.57, 95% CI 0.33–0.99, *p* = 0.047), and genus: *Eubacterium rectale* group (IVW OR = 0.37, 95% CI 0.18–0.77, *p* = 0.008), as well as the class: Lentisphaeria (IVW OR = 0.62, 95% CI 0.41–0.94, *p* = 0.026) and the order: Victivallales (IVW OR = 0.62, 95% CI 0.41–0.94, *p* = 0.026), exhibited a causal inclination towards reducing the risk of MM. No significant reverse causal relationship between gut microbiota and any of the MM SNPs was observed in the reverse-MR analysis. Furthermore, IVs did not exhibit significant heterogeneity or horizontal pleiotropy. The MR analysis underscored the correlation between the presence of MM and the gut microbiota.

### 3.3 Sensitivity analysis and pleiotropy identification

Sensitivity analysis was conducted to evaluated the robustness of the MR analysis and to identify potential IVs. The MR-Egger regression did not detect any heterogeneity among the 7 taxa, indicating the absence of horizontal pleiotropy. Furthermore, Cochran’s Q test revealed no heterogeneity, with all *p*-values above 0.05 (Supplementary Table S3). The forest plots displayed symmetrical results, further confirming the lack of heterogeneity in the data (Supplementary Figure S2). Moreover, the leave-one-out analysis demonstrated that removing any SNP did not significantly impact the outcomes (Supplementary Figure S3). The MR-PRESSO analysis identified no outliers with pleiotropic effects among the 7 taxa related to MM, with a global test *p*-value >0.05 (Table 1; Supplementary Table S4). Therefore, there was insufficient evidence of horizontal pleiotropy in the correlation between these microorganisms and MM. Additionally, PhenoScanner indicated that none of the 7 taxa had any SNP associated with potential confounding factors (Supplementary Table S5). The FDR corrected *p*-value is available in Supplementary Table S2. In conclusion, the sensitivity analyses in this study provide compelling evidence supporting a consistent causal relationship between gut microbiota and MM based on our MR analyses.

**Table 1:** Sensitivity analysis for significant and nominal significant estimates.

Exposure	Egger intercept	*p*val	*Q*	*p*-value	MR-PRESSO global test
Genus: *Eubacterium ruminantium* group	−0.045	0.432	14.646	0.621	0.616
Genus: Dorea	0.084	0.183	5.456	0.859	0.848
Class: Lentisphaeria	0.601	0.599	4.145	0.763	0.804
Order: Victivallales	0.060	0.599	4.145	0.762	0.809
Genus: Coprococcus1	0.047	0.519	14.971	0.133	0.185
Genus: RuminococcaceaeUCG014	0.002	0.974	8.141	0.615	0.544
Genus: *Eubacterium rectale* group	−0.048	0.605	6.699	0.569	0.628

## 4 Discussion

This study is the first to utilize MR in exploring the impact of gut microbiota on MM risk. Using the largest GWAS datasets available, our MR analysis uncovered a significant association between increased abundance of a specific genus *Eubacterium ruminantium* group and a higher risk of MM development. Furthermore, we identified six other gut microbiota that may potentially exert a beneficial impact on MM. This underscores the considerable contribution of gut microbiota in the progression of MM and offers valuable perspectives for future inquiries. The escalating mortality rate of MM in recent times can be attributed to its propensity to evolve into renal failure as the disease advances. Consequently, comprehending the etiology of MM and implementing proactive interventions to avert its onset assume paramount significance.

The gut microbiota assumes a crucial function in the assimilation and utilization of soluble fiber, fats, proteins, and vitamins. The prognosis of multiple myeloma is directly impacted by the consumption of nutrients. Additionally, in addition to participating in the process of nutrient assimilation, the gut microbiota also plays a role in activating signal transduction pathways, thereby stimulating immunity, and regulating the release of cytokines in intestinal epithelial cells (23). Over the past decade, the survival rate of most MM patients has increased due to the introduction of new substances such as proteasome inhibitors and immunomodulatory drugs, although MM remains largely incurable (24). Growing evidence suggests that changes in gut microbiota can influence cancer development through inflammation and complex host-microbiota interactions (25). However, a definitive causal relationship between gut microbiota and the pathogenesis of MM has yet to be established. Consequently, the findings of our study propose a comprehensive mechanism associated with nutrient preservation and present novel insights for the future treatment of MM.

The MR analysis demonstrated that the ruminantium group presents a potential risk for MM, thereby requiring additional mechanism studies to authenticate its functionality. However, a previous investigation found that the reduction in *Eubacterium ruminantium*, associated positively with SCFAs and antioxidant markers but negatively with inflammatory cytokines, could enhance the efficacy of chlorogenic acid in preventing alcoholic liver disease through the gut-liver axis (26). Subsequent research uncovered a significant increase in *Eubacterium rectale* in melanoma patients responding well to anti-PD1 immunotherapy, with higher levels of *Eubacterium rectale* associated with prolonged survival. The use of *Eubacterium rectale* significantly improved the effectiveness of anti-PD1 therapy and extended the lifespan of mice with tumors. Additionally, administering *Eubacterium rectale* resulted in a notable accumulation of NK cells in the tumor microenvironment as reported in reference (27). *Eubacterium rectale*, a butyrate producer, was found to reduce the incidence of primary gastrointestinal B cell lymphomas in humans while supporting the survival and growth of intestinal B cells (28). Increased effectiveness in treating MM was linked to higher levels of commensal microbiota affecting inflammatory responses through butyrate production. Similarly, better outcomes following hematopoietic stem cell transplantation were associated with increased gut microbiota diversity in individuals with hematologic malignancies. The interconnectedness of these findings suggests that commensal microbiota significantly influences survival rates, susceptibility to infections, disease recurrence, and the occurrence of graft-versus-host disease (GVHD) post-transplantation (29).

Ongoing basic and clinical studies are investigating the relationship between intestinal flora diversity and multiple myeloma. A retrospective study in China analyzed the intestinal microbiome composition and diversity in 40 newly diagnosed MM patients and 17 controls. The study revealed reduced intestinal flora diversity in the MM group compared to healthy controls, with a higher prevalence of butyrate-producing bacteria correlated with advanced MM (23). These findings imply that butyrate-producing bacteria like *Eubacterium rectale* could potentially help alleviate the harsh effects of chemotherapy, immunotherapy, and radiotherapy by inhibiting pro-inflammatory cytokines. Research also suggests that MM patients with elevated levels of the butyrate-producing bacterium *Eubacterium Hallii* were more likely to achieve a robust treatment response (30).

In the context of MM studies, the absence of specific microbiota groups was observed. Conversely, in different MR studies, a reverse association between the order: Victivallales and sepsis risk was identified (31). Additionally, a positive correlation has been observed between RuminococcaceaeUCG014 and the risk of asthma (32). A prospective study investigating pre-transplant dietary habits in 30 multiple myeloma patients who underwent autologous stem cell transplantation with melphalan 200 mg/m^2^ revealed suboptimal consumption of fiber, vegetables, and whole grains in comparison to national dietary recommendations. This dietary pattern may potentially influence the composition of the intestinal microbiome. Moreover, the study corroborated previous research indicating a substantial reduction in gut microbiome diversity following transplantation, particularly in the Ruminococcus genera, and proposed a potential link between these diversity alterations and the administration of intravenous antibiotics for the management of neutropenic fever (33).

The precise mechanisms through which other accumulated bacterial communities identified in the article influence the pathogenesis of MM are currently unknow. Previous research indicates that heightened mitochondrial function may potentially impact a specific microbiota associated with elevated levels of Dorea. Consequently, there was an elevation in the process of fatty acid oxidation, ultimately leading to the potential postponement of the progression of non-alcoholic steatohepatitis (34). The group subjected to the intervention exhibited an augmentation in the proportion of genus: Dorea, which consequently yielded enhancements in symptoms related to depression. Furthermore, the intervention demonstrated favorable impacts on depressive symptoms and induced alterations in the composition of the gut microbiota (35). While no statistically significant difference was observed, observational studies have not definitively established a direct link between the identified gut microbiota and MM progression. Microbiota-related metabolites such as SCFA, butyrate, and NAD+ may act as markers for MM progression, providing valuable insights for future research endeavors.

With the extended lifespan of multiple myeloma (MM) patients, there is growing evidence to examine the impact of dietary and lifestyle factors on the incidence and mortality rates of the disease. Dietary modifications can impact the makeup of the host microbiome, leading to the development of customized nutritional interventions to regulate the gut microbiome. Recent studies exploring the relationship between dietary habits, gut microbiota composition, and metabolic syndrome have indicated that the consumption of an animal-based diet is linked to a decrease in *Eubacterium rectale* and *Ruminococcus bromii*, which are involved in breaking down plant-based polysaccharides. Conversely, a plant-based diet has been shown to increase levels of short-chain fatty acids (SCFAs) like acetate and butyrate in fecal samples, while an animal-based diet results in higher concentrations of isovalerate and isobutyrate. Individuals adhering to a plant-based diet have a lower risk of multiple myeloma (MM) compared to those who consume meat (36). Studies suggest that a higher intake of fruits is associated with a reduced likelihood of developing monoclonal gammopathy of undetermined significance (MGUS) and progressing to MM (37). Those following a vegetable-rich diet, especially with cruciferous vegetables, exhibit higher levels of SCFAs, which are linked to a lower MM risk, possibly due to the presence of butyrate-producing bacteria like *Eubacterium rectale* (38). Whole grains have demonstrated a lower glycemic index and reduced levels of insulin and insulin-like growth factor-1 (IGF-1), both implicated in MM development. Consequently, consumption of a whole-grain diet is connected to a decreased MM risk (39, 40). Some studies suggest that probiotics capable of producing butyrate may enhance gut bacterial diversity and possess anti-inflammatory properties (41, 42). While probiotics are a popular method for modulating the gut microbiome, there is a lack of comprehensive data on the effects of probiotic supplementation in MM patients. Further clinical investigations are essential to thoroughly understand the potential risks and advantages of probiotics on the microbiome as well as MM risk and prognosis. These findings highlight a high level of adaptability, indicating the possibility of reducing the risk of multiple myeloma or improving treatment outcomes. Dysbiosis has been identified as a key characteristic of multiple myeloma, emphasizing the need for comprehensive cohort studies that consider simultaneous assessments of dietary patterns and microbiome compositions.

This research offers numerous benefits. To ascertain the causal relationship between gut microbiota and MM, we conducted a MR analysis. The objective of this analysis was to mitigate the impact of confounding variables and reverse the causation direction, thereby enabling accurate causal inference. The genetic variations of gut microbiota were obtained from the most comprehensive publicly available GWAS meta-analysis, ensuring the reliability and robustness of the instruments employed in our MR analysis. The MR-PRESSO and MR-Egger regression intercept term tests were employed to detect and mitigate any potential occurrence of horizontal pleiotropy. To mitigate bias, a two-sample MR design was utilized, and FDR analysis was conducted (16). When interpreting the findings, it is important to consider the limitations of this study. Despite not meeting the Bonferroni-adjusted significance threshold, the MR analysis conducted in this study aimed to test epidemiologically established associations that are supported by physiological evidence.

Furthermore, it is important to acknowledge that the analysis was based on summary statistics rather than raw data. It is crucial to recognize the study’s limitations, particularly when extrapolating findings to different racial groups due to significant differences in ancestral backgrounds between individuals of European descent (78%) and other ancestries (22%). Population stratification remains a concern, potentially limiting generalizability to non-European populations. To enhance the relevance of future studies, it is advisable to investigate the gut microbiota-MM association in diverse populations from Europe and beyond (31). Incorporating additional genetic variations as instrumental variables during sensitivity analyses is recommended to identify and address potential horizontal pleiotropy. The SNPs used in the analysis did not meet genome-wide association study significance thresholds (*p* < 5 × 10^−8^). FDR correction was employed to minimize the risk of false positives. The reverse MR analysis may have been influenced by weak instrumental bias due to the limited MM dataset sample size, making it impossible to rule out reverse causation entirely. Our study did not encompass all gut microbiota identified in previous research, possibly due to a limited number of genetic loci from GWAS. It is vital to acknowledge that our instrumental variables may not be optimal, potentially reducing the statistical power of our MR study. Increasing the sample size for analysis is essential to address this issue effectively.

In conclusion, this research represents a preliminary exploration of applying Mendelian randomization as a technique to investigate the connections between gut microbiota and MM. Future mechanistic and clinical investigations into the influence of microbiota on MM could derive substantial insights from the results of this study.

## Data availability statement

The original contributions presented in the study are included in the article/Supplementary material, further inquiries can be directed to the corresponding authors.

## Ethics statement

The manuscript presents research on animals that do not require ethical approval for their study.

## Author contributions

C-YZ: Writing – original draft, Writing – review & editing. DZ: Conceptualization, Software, Writing – original draft, Writing – review & editing. W-RS: Data curation, Project administration, Writing – review & editing. HT: Funding acquisition, Project administration, Software, Writing – review & editing. BT: Methodology, Software, Writing – review & editing. LH: Formal analysis, Methodology, Writing – review & editing. WH: Data curation, Writing – review & editing. Y-YG: Formal analysis, Writing – review & editing. M-YL: Formal analysis, Writing – review & editing. W-TX: Methodology, Writing – review & editing. SG: Investigation, Writing – review & editing. G-XG: Funding acquisition, Supervision, Writing – review & editing, Writing – original draft.

## Footnotes

^1^ https://mibiogen.gcc.rug.nl

^2^ https://finngen.gitbook.io/documentation/v/r5/

^3^ http://www.phenoscanner.medschl.cam.ac.uk
